# Supplementary material for: Are carbon emissions trading and green financial instruments synergistic? -Comprehensive quantitative research based on content analysis
Source: PLoS One. 2024 Mar 7;19(3):e0298601. doi: 10.1371/journal.pone.0298601 (PMC10919601; doi:10.1371/journal.pone.0298601)
Supplement: S1 Data — (ZIP) [file pone.0298601.s001.zip › Data/地级市Z_F工作报告.zip!/╘╞─╧/└Ñ├≈╩╨/└┤╫╘╣1⁄2╓┌║┼í╛┬φ┐╦╩2╛▌═°í┐_2014.docx]

各位代表：

　　我代表市人民政府向大会报告工作，请予审议，并请市政协委员和列席人员提出意见。

　　一、攻坚克难，团结奋进，经济社会发展成效显著

　　2013年，是我市发展进程中极不平凡的一年。面对错综复杂的国内外形势和集中显现的社会矛盾，在省委、省政府和市委的坚强领导下，在市人大、市政协的监督支持下，市政府认真贯彻落实党的十八大精神，牢牢把握发展第一要务，坚定不移稳增长、转方式、调结构、促改革、惠民生，沉着应对困难和挑战，妥善处理热点难点问题，经过全市人民的共同努力，保持了全市经济较快发展、社会和谐稳定。

　　（一）结构调整不断优化，经济平稳较快增长。预计全市地区生产总值3450亿元，增长13%，三次产业结构调整为5.1∶45∶49.9；地方公共财政预算收入450.8亿元，增长19.1%；规模以上固定资产投资2930亿元，增长25%；社会消费品零售总额1700亿元，增长14%；居民消费价格上涨3.9%；城镇居民人均可支配收入28344元，农民人均纯收入9269元，分别增长12.3%、15.3%；万元生产总值能耗下降3.9%。市十三届人大四次会议确定的目标任务基本完成。

　　（二）产业建设扎实有力，发展后劲进一步增强。以“8185”产业培育提升为抓手，加快重点产业培育。粮食产量123万吨，烤烟效益显著提升，都市农庄试点取得成效。规模以上工业企业925户，增加值增长12%。开工亿元以上工业项目80项，竣工55项。园区规模以上工业增加值占全市的88%，杨林工业园区升级为国家级园区。14个泛亚商贸物流中心加快建设，主城商品交易市场有序搬迁，建成51个菜市场和9条美食街区。全市接待游客5600万人次，增长19%；旅游总收入516亿元，增长21%。

　　（三）社会事业全面进步，民生福祉不断增进。深入实施“十大民生工程”，民生支出占地方公共财政预算支出的70%。城镇新增就业12.4万人、再就业3.5万人，城镇登记失业率2.67%。扶持1.4万人成功创业。培训农村劳动力34万人，转移就业23.6万人。城乡居民基本养老保险、医疗保险参保覆盖率分别达96.7%、97.8%。城镇职工基本养老、失业、工伤、生育保险参保覆盖率均提高到96%以上。城乡低保标准平均增幅分别达15.2%和17%。建成保障性住房4.47万套。完成26所高（完）中新建、迁建、改扩建项目。学前教育毛入园率提高到96.5%，义务教育巩固率保持在99%以上，随迁子女义务教育阶段就读人数达20万人。61万贫困学生得到资助，36万农村学生在营养改善计划中受益。基本公共卫生服务项目持续推进，覆盖城乡居民482万人次。市第一人民医院北市区医院、市中医院呈贡新区医院试营业。民营医院达171家，床位1.2万张。人口自然增长率5.6％。。完成广播电视“村村通”直播卫星覆盖工程。新增国家级文物保护单位3项、省级非物质文化遗产保护名录项目22项。市体育学校暨全民健身中心基本建成，成功举办高原国际半程马拉松赛和市第十届民运会。加强矛盾纠纷排查调处，积极应对网络舆情。完善社会治安防控体系，严厉打击各类违法犯罪，群众安全感不断增强。食品药品安全形势稳中向好，覆盖全市的肉菜流通追溯体系基本形成。强化安全隐患排查，安全生产形势总体平稳。双拥工作取得新成效，全市民族团结、宗教和睦。

　　（四）城市建设加快推进，载体功能得到提升。编制完成各类规划54个，基本实现市域总体规划、县（市）区中心区控制性详规、环滇主城四片城市形象设计“三个全覆盖”。武昆高速、绕城高速西北段、阳宗海西部快线一期建成通车，南连接线、功待、黄马高速等项目顺利推进，呈澄高速等9个项目开工。地铁1、2号线首期工程南段通车试运营，3号线工程加快推进。基本完成5条铁路征地拆迁，新火车南站开工建设。中缅天然气管道干线全线贯通。完成以“六路一江”为重点的景观亮化设施建设，综合整治北京路等31条城市道路、9条微循环道路和17个交通节点。建成70个立体停车场。主城公共交通机动化出行分担率47.3%。完成牛栏江应急备用水源补给工程，清水海引水工程每天可向城市供水30万立方米。以城中村为主的城市更新改造取得新成效。呈贡新区公共服务设施及城市综合体项目加快实施。

　　（五）滇池治理力度加大，生态环境持续改善。围绕滇池治理六大工程，启动实施“三年行动计划”，完成投资88.6亿元。埋设截污管网153公里。第九、十污水处理厂及主城10个调蓄池通水运行。拆除环湖路内大棚1055亩、防浪堤43公里，建设生态堤埂23公里，增加滇池水域面积11.5平方公里。完成盘龙江清淤除障30万立方米。滇池外海水体透明度上升4.88%，草海、外海综合营养状态指数分别下降0.86%和1.2%。阳宗海湖体水质保持稳定。全市集中式饮用水源地水质稳定达标。滇池流域“五采区”植被修复8184亩，市级退耕还林20.4万亩，新增城市绿地1028公顷，森林覆盖率48%，荣获“国家森林城市”称号。完成节能减排重点项目196个，1000辆节能与新能源汽车投入运行。主城区空气质量优良天数达333天。

　　（六）县域经济协调推进，城乡发展加速融合。各县（市）区经济实力不断增强，五华、官渡地区生产总值预计均超过700亿元，6个县区被评为全省县域科学发展先进县。保护耕地和基本农田，节约集约用地，低丘缓坡土地综合开发利用试点16万亩。“农转城”28.7万人，全市城镇化率68%。完成农村危房改造及地震安居工程1.8万户。行政村公路路面硬化450公里。新增城乡公交线路11条，公交“镇镇通”实现全覆盖、行政村覆盖率94.5%。建成68件农村抗旱应急工程、41件水源和病险水库除险加固工程，完成8万件“爱心水窖”。建成142个新农村省级重点村和30个市级示范村。145个重点贫困行政村基本实现“一通六有”，解决6万农村贫困人口温饱问题。

　　（七）改革开放步伐加快，发展活力日益显现。进一步下放市级审批权限10项。“营改增”试点启动实施。落实促进民营经济发展政策措施，民营经济预计实现增加值1560亿元，增长14%以上。完成农村集体土地所有权确权颁证。教育体制改革试点取得阶段性成效。

20所县级公立医院被列为国家改革试点，药品实行零差率销售。第五次荣获“全国科技进步先进市”称号。加强和改进招商引资工作，实际利用外资17.9亿美元，增长13.2%；引进市外到位资金1297亿元，增长4.8%。实现进出口贸易总额170亿美元，增长18%。投融资体制改革取得新成绩，政府债务风险可控。新引进25家金融机构。缔结国际友城3个，孟加拉国在昆设立领事馆。圆满完成首届中国—南亚博览会暨第21届昆交会、第13届亚洲艺术节筹办和服务保障工作。

　　（八）政府建设不断加强，行政行为进一步规范。加强政府立法工作，制定、修订政府规章7件，提请市人大制定、修订、废止地方性法规11件。自觉接受市人大法律监督、工作监督，主动接受市政协民主监督，认真听取各民主党派、工商联和无党派人士意见，办理市人大代表建议293件、市政协提案546件。发挥政府网站、政务微博作用，广泛接受社会监督。严格落实中央“八项规定”和省、市相关要求，切实改进作风、文风和会风，“三公”经费压缩19.6%。认真执行廉洁从政各项规定，严肃查处一批违法违纪案件，廉政建设取得新成效。

　　各位代表！奋斗充满艰辛，成绩来之不易。我们取得的成绩，是省委、省政府和市委正确领导的结果，是全市人民团结奋斗、辛勤工作的结果。在此，我代表市人民政府，向全市人民，向人大代表、政协委员，向各民主党派、工商联、人民团体和社会各界人士，向中央省属单位、驻昆部队和武警官兵，向所有关心支持昆明建设发展的海内外朋友，表示衷心的感谢！

　　在总结成绩的同时，我们也清醒地看到，我市经济社会发展还面临不少困难和问题，主要表现在：全市经济总量不大、质量不高，转方式、调结构任务繁重；行政审批程序复杂、效率不高，监管不到位，公共产品提供还不够；城乡、区域之间发展不平衡；城市规划建设管理水平不高，对历史文脉传承保护利用不够，市容市貌脏乱差、交通拥堵等问题突出；资源环境约束加剧，生态修复难度大，滇池治理任重道远；社会矛盾多发易发、交错叠加，一些热点难点问题尚未得到有效解决；少数部门和公务员精神懈怠、作风漂浮、能力不足，“四风”问题仍不同程度存在。对此，我们必须高度重视，认真加以解决。

　　二、稳中求进，奋力拼搏，推动经济社会持续健康发展

　　2014年是深入实施“十二五”规划承前启后的关键一年。我们必须充分认识发展是解决所有问题的关键，坚持把发展作为第一要务，认真落实国家和省的总体要求，按照市委十届四次全会的决策部署，扎实抓好各项工作，促进经济社会又好又快发展。

　　今年政府工作的总体要求是：以党的十八届三中全会、中央经济工作会和习近平总书记系列重要讲话精神为指导，牢固树立进取意识、机遇意识、责任意识，以稳中求进、改革创新为主旋律，坚持科学发展，着力转方式、调结构，保持经济的合理健康发展，进一步解放思想，全面深化改革，激发市场活力，更加注重改善民生，更加注重社会事业的发展和生态文明建设，更加注重社会和谐稳定，加快建设美好幸福新昆明，为在全省率先全面建成小康社会打下坚实基础。

　　今年经济社会发展主要预期目标建议为：地区生产总值增长12%左右，地方公共财政预算收入增长13%以上，规模以上固定资产投资增长20%以上，社会消费品零售总额增长13%以上，城镇居民人均可支配收入和农民人均纯收入分别增长11%和13%以上，居民消费价格涨幅控制在4%以内，万元生产总值能耗下降3.7%，城镇登记失业率控制在4%以内，人口自然增长率控制在6％。以内。

　　今年，我们将重点做好以下六个方面的工作：

　　（一）加快产业转型升级，不断壮大经济实力

　　推动经济快速发展。强化产业拉动，深入推进产业建设年活动，努力构建“8185”+X现代产业体系，三次产业增加值分别增长5%、13.5%和12%以上。强化投资拉动，实施重点项目220项，投资775亿元，其中，基础设施及民生项目90项，投资349亿元；产业项目90项，投资426亿元；推进前期项目40项。加强项目土地储备，严格执行净地出让制度，全面实施网上交易，完善项目引进评审和退出机制，预留大项目用地空间。整合财政资金，实施财政贴息、风险补偿、奖励引导等措施，支持实体经济发展。强化消费拉动，巩固发展餐饮、商贸等传统消费，积极培育文化、信息、休闲等消费热点，加快国家电子商务示范城市建设，大力发展网购网销等新兴消费业态，引导住房、汽车等大宗消费健康发展。

　　提升经济发展质量。坚持工业打头阵，加快工业园区规划建设，改造提升有色金属、化工、黑色金属及建材等传统工业，加速发展生物医药、非烟轻工等消费品工业，培育石油炼化及天然气、新能源汽车、新材料、信息、节能环保等新兴产业。有效化解过剩产能，加快工业企业“去库存化”。新开工亿元以上工业项目60项、竣工50项，规模以上工业企业达980户。按照“产城一体化”要求，完成全市工业布局规划调整。积极发展以服务业为主的城市经济，打造一批城市综合体，推进以东风广场片区为中心的中央商务区、老螺蛳湾片区等次级商务区建设，新增总部企业20户、楼宇面积160万平方米。改造提升城市传统商圈，加强社区商业设施建设，培育地铁商业，继续实施“万村千乡市场工程”。抓住云南沿边金融综合改革试验区建设契机，强化金融产业园区建设，打造昆明区域性国际金融中心。加强以螺蛳湾国际商贸城为重点的商贸服务业聚集区建设，加快主城商品交易市场整治搬迁。推进泛亚商贸物流中心建设，重点培育10家物流企业集团。以昆曲绿色经济示范带建设为重点，发展高原特色农业。稳定粮食生产，巩固烤烟支柱，提高蔬菜、花卉效益，大力发展畜牧业、林产业和农产品加工业。加快石林台湾农民创业园、嵩明现代农业科技示范园、斗南国际花卉园等农业园区建设，着力培育龙头企业、都市农庄、家庭农场等新型经营主体。注重农产品质量安全，无公害农产品、绿色食品、有机农产品和农产品地理标志认证30个。壮大民营经济，新增1万户小微企业，培育50户企业升级为规模以上企业，增加值突破1700亿元。

　　加强科技创新驱动。深入推进国家创新型试点城市建设，全社会研发投入强度达1.77%，科技成果转化率达41.4%。发挥大型企业创新骨干作用，实施60项高新技术产业化项目，认定40家高新技术企业。新增企业技术中心40家以上、省级名牌产品20个以上。培育3个产业技术创新战略联盟和5个院士工作站，新增3个博士后工作扶持站。开展25项“科技惠民”计划项目。新增知识产权试点示范单位10家。启动国家级文化和科技融合示范基地建设。

　　（二）着力保障改善民生，提高社会发展水平

　　提供更多的就业创业机会。鼓励多渠道多形式就业，促进以高校毕业生为重点的青年就业和农村转移劳动力、城镇困难人员、下岗职工、退役军人就业。新增城镇就业10万人，农村劳动力转移培训20万人、转移就业15万人。实施“离校未就业高校毕业生就业促进计划”，确保实名登记的高校毕业生就业率90%以上、困难毕业生100%就业。扶持自主创业，创建市级大学生创业示范园5个、农业创业示范乡村20个。

　　实现更高水平的社会保障。推进社会保险全民覆盖，逐步提高社会保险待遇。完善落实社会救助、社会保障标准和物价上涨联动机制，城乡低保、五保供养标准提高15%以上。完善社会养老服务体系，支持社会力量兴办养老机构，新增养老床位4500张，启动建设60个居家养老服务中心。以城市棚户区改造为重点，开工建设保障性住房2.6万套，做好保障房分配、配套设施建设和运营管理。完成农村危房改造及地震安居工程2.5万户。

　　提供更优质的教育资源。着力扩大公办和普惠性民办学前教育资源，学前教育毛入园率达97%以上。实施义务教育“增量扩优”工程，巩固提高普通高中“强基提质”成果，高中阶段毛入学率提高到92%以上。完善现代职业教育体系，优化提升民办教育。深化平安校园创建工作，加快实施校舍改造工程，推进农村义务教育阶段学生营养改善计划。

　　建设更高标准的健康服务体系。着力解决“看病难、看病贵”问题，新增病床3000张，每千人拥有执业（助理）医师数3.5人。落实基本药物制度，减轻群众就医负担。市第一人民医院北市区医院、市中医院呈贡新区医院全面投入使用。开展社区医生和居民契约服务试点，完善社区双向转诊医疗模式。加强疾病防控和妇幼卫生保健工作，提高突发公共卫生事件处置能力。

　　营造更加和谐的社会环境。强化“平安昆明”建设，健全立体化防控体系，严厉打击各类违法犯罪。加强网络安全防控，维护良好网络秩序。健全重大决策社会稳定风险评估制度，完善应急处置机制，有效应对和妥善处置突发事件。推进和谐社区建设，确保90%以上社区创建达标。搞好粮食流通储备，抓好“米袋子”、“菜篮子”工程，履行行政首长负责制，确保主要农副产品市场价格总体平稳，让市民买菜更方便。加强食品药品监管，保障食品药品安全。完善安全生产网格化监管机制，抓好重点行业和领域安全专项整治，严防重特大安全事故发生。健全防灾减灾体系，创建5个全国综合减灾示范社区和3个全国地震安全示范社区。做好森林防火和防汛抗旱工作。加强国防后备力量建设，推动军民融合深度发展，争创第十届全国双拥模范城。做好民族宗教工作。

　　（三）强化生态文明建设，努力打造美丽春城

　　以更大的举措治理滇池。深入实施滇池治理“三年行动计划”，完成38个治理项目、投资126亿元以上。突出抓好彻底截污、水体置换和生态建设，强化“河（段）长责任制”和“河道三包”责任制，确保国家考核的16条主要入湖河道水质达标率70%以上。推进第十一、十二污水处理厂及底泥疏浚三期等工程，完成主城污水处理厂尾水外排、污泥处置及牛栏江滇池补水出水口瀑布公园等项目。建设11个环湖湿地生态公园，抓好滇池流域“五采区”植被恢复和郊野森林公园建设，努力实现滇池流域面山绿化全覆盖。

　　下大力气保护好生态环境。新造林35万亩以上，市级退耕还林20万亩，森林覆盖率49%以上。做好高速公路沿线、城市道路和街头绿化，完善三级公园绿地体系，提升“十大公园”品质，保护恢复“三山一水”生态格局和特色风貌。抓好牛栏江、南盘江、螳螂川等流域水环境综合整治，加强饮用水源区和生态脆弱区保护。深入开展生态县、乡（镇）、村和绿色学校、社区创建工作，加强自然保护小区建设。严格环保执法，严惩破坏生态环境行为。巩固提升“四创两争”成果，启动创建国家生态园林城市。

　　全力提高低碳发展水平。推进低碳城市试点工作，实施一批低碳、节能重点工程，创建一批循环经济示范县区、园区和企业。推广应用新能源与可再生能源，加快淘汰落后产能，推进重点行业企业清洁生产。推广绿色建筑，主城五区、三个开发（度假）区和阳宗海风景名胜区新建建筑全部执行国家绿色建筑标准。加强水资源管理，严格区域用水总量、用水效率及水功能区限制纳污“三条红线”指标控制。加强PM2.5监测，整治汽车尾气、工地扬尘污染，主城区空气质量优良率达85%以上。

　　（四）着力提升城市品质，建设世界知名旅游城市

　　突出规划引领作用。围绕建设世界知名旅游城市，按照“一湖四片、一主四辅、一城多点”空间布局，完成总规修订和报批工作，抓好控制性详规、分区规划、片区规划、小区规划和单体设计。统筹地上、地下空间开发利用，编制完成综合交通体系规划、市域与滇池流域永久生态保护规划和城市排水（雨水）防涝综合规划。推进建设规划、土地利用规划和林地保护利用规划“三规合一”。注重传承历史文脉，强化城市色彩和空间形态控制，突出春城特色的城市设计，彰显少数民族文化的城市风貌。加强规划监督管理，增强规划的约束力和执行力。

　　加快城市基础设施建设。深入实施“3015行动计划”，完善“一湖四片”、“一主四辅”道路网络，初步建立环滇池立体交通体系。加快绕城高速东南段、呈澄高速、黄马高速、金东大桥、寻倘、东倘、禄倘等项目建设，完成老昆洛路朱家村至呈贡段提升改造，长嵩大道、南连接线建成通车。加快新火车南站及外部配套基础设施建设。开工建设地铁1号线延长线和2、3、6号线二期工程，1、2号线首期工程全线开通试运营。完善人防、电力、燃气、排水防涝、垃圾污水收集处理等市政设施。推进“六大片区”建设，提速呈贡新区基础设施建设，抓好市文化馆、中心图书馆、文化传媒中心等项目建设。抓住云南打造航空强省的机遇，加快空港经济区建设。

　　提升城市管理水平。千方百计缓解交通拥堵，实施城市干道、支次路网和微循环系统建设，新增停车泊位5万个，完善交通标识等设施，强化交通秩序管理，实行主城大货车限行。加快创建国家“公交都市”示范城市，公共交通机动化出行分担率达52%。抓好道路桥梁安全管理，加强城市照明和景观亮化管护。强化文明施工，整治渣土运输，严格规范道路开挖。坚持先建后拆、平稳拆迁、和谐拆迁，推进中心城区旧厂旧村旧城更新改造，坚决制止违法加层、无序建房和违规建设商品交易市场。开展“城市清洁工程”行动，实行“路长制”，集中整治背街小巷、城中村、城郊结合部和城市主要出入口环境卫生，整治占道经营，防止“脏乱差”现象反弹。深化节水型城市建设。加快科学调度水源，确保城市供水安全。推进“三网融合”和第四代移动通讯网络建设。加快打造“智慧城市”。

　　促进文化昆明建设。加强文化传承保护利用，推进全国第一次可移动文物普查，实施15个不可移动文物保护工程。抓好非物质文化遗产精品项目建设，完成第四批国家级名录申报及文庙恢复性修建项目一期工程。鼓励民营文艺院团、演出市场中介和娱乐企业连锁发展，打造一批骨干文化企业和知名文化产业品牌。推进“户户通”建设，启动地面数字电视覆盖工程，实现基层公共文化站（室）建设达标率90%以上。加快“七彩云南全民健身工程”建设。推进文旅融合，构建“一核一带一群四片九区”旅游产业发展格局。加快“十大景点”建设，抓好古滇国文化旅游名城等一批旅游项目。开展“十个一”旅游信息化建设和宣传营销活动，拓展国内外旅游市场。

　　（五）统筹城乡融合发展，加快城乡一体化进程

　　壮大县域经济实力。根据各地资源禀赋和发展水平，加强对县域经济发展的分类指导，完善落实政策措施，培育主导产业，推动县域经济走特色化、差异化道路，支持资源型城市转型发展。做好扩权强县、扩权强镇工作，凡属基层管理更方便、更有效的经济社会管理事项一律下放。推进晋宁撤县设区和嵩明、宜良撤县设市工作。加快县城和小城镇开发建设，打造一批工业强镇、商贸重镇、旅游旺镇和历史文化名镇。稳步推进城镇上山，完成“农转城”10万人，全市城镇化率达69%。

　　加强农村基础设施建设。续建3件中型、14件小（一）型水源工程，新开工1件中型、3件小（一）型水库和40件病险水库除险加固工程。建设“五小水利”工程5万件，解决10万农村人口饮水安全问题。加强小型农田水利重点县建设，推进灌区续建配套和节水改造项目建设。改造中低产田20万亩。完成行政村公路路面硬化450公里，加强农村道路管养。新建气象公共信息电子显示屏1800块。

　　加快建设美丽乡村。开展农村环境综合整治，改善农村人居环境，注重保留村庄自然文化风貌，遏制农村建房的随意性、零散性和无序性，建设“三清四美三宜”美丽乡村。以安宁南部、呈贡万溪冲片区和市域高速公路沿线为重点，打造9条美丽乡村示范带，建成70个省级美丽乡村和30个市级美丽乡村示范村。

　　全力打好扶贫攻坚战。深入实施扶贫攻坚三年行动计划，启动129个重点贫困行政村“一通六有”建设，实施省级16个行政村、市级200个自然村整村推进，做好4个整乡推进续建工作。抓好东川和乌蒙山连片特困地区、少数民族地区发展与扶贫攻坚，加快倘甸扶贫开发综合实验示范区建设，易地搬迁1400人。

　　（六）致力扩大对外开放，培育合作竞争新优势

　　推进对外贸易平稳发展。优化外贸结构，力争进出口贸易总额达103亿美元。扩大先进技术设备、关键零部件和重要原材料进口。加大对高新技术、生物制药产品出口扶持力度，提高机电、有色冶金和农产品等优势特色产品出口规模。加快打造国家级和省级服务外包示范城市，推进服务外包产业园区建设，支持文化、金融保险等服务产品出口。鼓励企业和个人“走出去”，开展并购投资、证券投资和联合投资，承揽境外大工程、大项目。做好昆明综合保税区、国家级国际贸易中心城市申报建设工作。

　　提升招商引资质量。围绕“8185”产业培育提升和“3015”行动计划，依托各类产业园区，实行领导招商、团队招商、以商引商、产业链招商，确保实际利用外资17.9亿美元以上，引进市外到位资金1250亿元以上。以长三角、珠三角、环渤海区域及港澳台、东盟、南亚为重点，加强与世界500强、行业领先企业和高科技企业的合作。提高招商引资实效，改进考核办法，把项目资金到位率、产出率、集聚率、就业率、土地集约率作为重要指标，强化业绩评价、跟踪问效，促进项目落地、竣工达产。

　　加强区域交流合作。拓展与东南亚、南亚地区的合作领域，提升与泛珠三角、成渝、北部湾等区域的合作水平，积极参与长江经济带建设。主动融入滇中城市经济圈，配合省做好滇中产业聚集区建设，重点开发建设好东区。扩大与边境州市合作，大力发展口岸物流和保税物流。依托南博会、昆交会、农博会等平台，拓展对外合作领域。巩固发展国际友城关系。

　　三、积极探索，大胆实践，开创全面深化改革新局面

　　今年是全面贯彻落实党的十八届三中全会精神、全面深化改革的第一年。我们必须把改革创新贯穿于经济社会发展各领域各环节，抓住方向明、见效快的改革，科学制定实施方案，先行先试、稳扎稳打，大胆创新、重点突破，以改革促发展、促转方式调结构、促民生改善。

　　（一）突出抓好经济领域改革

　　发挥市场在资源配置中的决定性作用，深化经济体制改革。加强国有资产监督管理，推进国有企业改革，健全国有资本管理体制，推动国有资产证券化，加快市交投公司、滇池水务股份公司上市进程。鼓励国有、集体与非公有资本交叉持股，加快发展混合所有制经济。深化投融资体制改革，优化政府性项目资源配置，构建规划、国土、土储、属地政府和投融资公司的联动协调机制。坚持“谁举债、谁偿还、谁承担责任”，建立政府债务管理长效机制，严控新增债务，逐步化解存量债务，有效防控债务风险。优化财政支出结构，改进预算管理制度，建立跨年度预算平衡机制。合理划分市级和县（市）区事权与支出责任，完善一般性转移支付制度。推进资源性产品价格改革，完善资源要素价格形成机制。加强公共资源交易市场体系和电子化交易平台建设，推动土地使用权、矿业权等进入公共资源交易中心交易。建立健全公益性服务企业价格补贴机制。推进工商登记制度便利化改革，制定负面清单，实行非禁即入，促进非公经济健康发展。

　　（二）加快新型城镇化改革

　　深化农村产权制度改革，加快农村土地承包经营权、集体建设用地使用权和房屋所有权确权登记发证，健全市、县、乡三级农村产权流转交易服务体系，稳妥推动地权、林权、财产权流转租赁、抵押融资。加快建立城乡统一的建设用地市场，探索农村集体经营性建设用地出让、租赁、入股办法，逐步实现与国有土地同等入市、同权同价。研究制定被征地农民参与土地市场化分配的措施。深化集体林权制度配套改革，开展农村水价改革试点。放开建制镇和小城市落户限制，有序推进农业转移人口市民化，逐步实现城镇基本公共服务常住人口全覆盖。

　　（三）切实转变政府职能

　　处理好政府与市场、社会的关系，加大行政审批制度改革，把该放的权力放到位，把该管的事务管好。推行权力清单制度，依法公开权力运行流程，规范政府权力行使。实行投资项目技术审查和行政审批相分离，切实解决审批难、落地难问题。推进政府机构改革，完成卫生、人口和计划生育、食品药品监管、工商质监行政管理体制调整。深化事业单位分类改革。改革社会组织登记制度，激发社会组织活力。推进政府购买公共服务，对事务性管理服务，以合同、委托等方式向社会购买。依据主体功能区划分，科学确定绩效考核标准，完善发展成果考核评价体系。

　　（四）深化生态文明体制改革

　　完善资源有偿使用和生态补偿制度，健全生态功能区财政转移支付制度，加大对松华坝水库、云龙水库、清水海等集中式饮用水源保护区的反哺力度。加快建立滇池流域生态保护机制和环滇池区域开发建设机制。改革生态环保管理体制，严格执行环评限批，科学划定生态保护红线。建立主要污染物排放权交易制度、环境污染联防联控及预警机制，推进数字环保建设，加强环境保护信息公开和公众参与。

　　（五）全面推进民生事业改革

　　深化教育领域改革，加快义务教育阶段学校标准化建设，推动优质学校跨区域发展，实行学区化管理。推进招生制度改革，制定异地中考政策和外来随迁子女义务教育阶段就学管理办法。深化文化体制机制改革，鼓励文化企业跨地区、跨行业、跨所有制兼并重组。采取政府采购、项目补贴、定向资助、贷款贴息等形式，推动公共文化服务社会化发展。强化政府促进就业责任，建立经济增长与扩大就业联动机制，完善创业扶持政策。规范招人用人制度，消除影响平等就业的制度障碍和就业歧视。推进城乡社会保障一体化，完善社会保险衔接转续机制。深化医疗保障、医疗服务、公共卫生、药品供应监管体制综合改革，完善城乡居民大病保险、保障和救助机制，逐步提高城乡居民医保财政补助水平。深化公立医院改革，建立现代医院管理制度。鼓励社会办医，构建多元化医疗卫生服务体系。加大计划生育家庭奖励扶助力度，落实一方是独生子女的夫妇可生育两个孩子的政策。

　　（六）大力创新社会治理

　　发挥政府在社会治理中的主导作用，增强基层组织社会治理和维护稳定的能力，引导社会各方有效参与，建立政府与社会力量联动互补的社会治理和公共服务网络。创新有效预防和化解社会矛盾的体制机制，建立畅通有序的诉求表达、矛盾调处、权益保障机制。推行网上受理信访、部门联合接访和市县乡三级视频联合接访，维护群众合法权益。提升公民法律意识和道德素质。加强社会诚信体系建设，完善政府、企业和个人信用体系，营造良好信用环境。

　　各位代表！建设一个高效廉洁、务实为民的政府，是全市各族人民的期望，是做好今年各项工作的保证。我们将坚持依法治市，深化“法治昆明”建设，坚决维护宪法法律权威，自觉接受市人大及其常委会的法律监督、工作监督和市政协的民主监督，高度重视社会监督和新闻舆论监督。健全重大行政决策公示、咨询、听证等制度，提高决策的科学化、民主化、法制化水平。我们将深入贯彻落实中央“八项规定”，着力解决“四风”突出问题。扎实有效开展好群众路线教育实践活动，认真践行“昆明干部精神”，真抓实干、取信于民。我们将认真落实中央惩治和预防腐败五年规划，履行党风廉政建设责任制，规范经费审批管理和报销制度，严控“三公”经费支出和楼堂馆所建设，严格公务接待标准，严禁公款吃喝、多占办公用房及乱发奖金、实物、补贴，落实婚丧喜庆、公务用车、公共场所禁烟等各项规定，严肃查处各类违法违纪案件，营造清正、清廉、清明的良好政风。

　　各位代表！昆明的发展翻开了崭新一页，我们正站在新的历史起点上。让我们紧密团结在以习近平同志为总书记的党中央周围，在省委、省政府和市委的坚强领导下，凝心聚力、团结拼搏、埋头苦干，共同谱写好“中国梦”的昆明篇章！
